# A Brain Anti-Senescence Transcriptional Program Triggered by Hypothalamic-Derived Exosomal microRNAs

**DOI:** 10.3390/ijms25105467

**Published:** 2024-05-17

**Authors:** Josefa Krarup, Lucas Araya, Felipe Álvarez, Daniel A. Bórquez, Pamela J. Urrutia

**Affiliations:** 1 Laboratory of Cell Signaling & Bioinformatics, Center for Biomedical Research, Faculty of Medicine, Universidad Diego Portales, Ejército Libertador 141, Santiago 8370007, Chile; josefa.krarup@mail.udp.cl (J.K.); felipe.alvarez1@mail.udp.cl (F.Á.); 2 Department of Biology, Faculty of Sciences, Universidad de Chile, Santiago 7810000, Chile; lucas.araya@ug.uchile.cl; 3 Laboratory of Resilient Aging, Institute for Nutrition & Food Technology (INTA), Universidad de Chile, El Líbano 5524, Santiago 7830490, Chile; 4 Geroscience Center for Brain Health and Metabolism, Santiago 7800003, Chile

**Keywords:** aging, miRNAs, bioinformatics, exosomes, transcriptomics, neural stem cells

## Abstract

In contrast to the hypothesis that aging results from cell-autonomous deterioration processes, the programmed longevity theory proposes that aging arises from a partial inactivation of a “longevity program” aimed at maintaining youthfulness in organisms. Supporting this hypothesis, age-related changes in organisms can be reversed by factors circulating in young blood. Concordantly, the endocrine secretion of exosomal microRNAs (miRNAs) by hypothalamic neural stem cells (htNSCs) regulates the aging rate by enhancing physiological fitness in young animals. However, the specific molecular mechanisms through which hypothalamic-derived miRNAs exert their anti-aging effects remain unexplored. Using experimentally validated miRNA–target gene interactions and single-cell transcriptomic data of brain cells during aging and heterochronic parabiosis, we identify the main pathways controlled by these miRNAs and the cell-type-specific gene networks that are altered due to age-related loss of htNSCs and the subsequent decline in specific miRNA levels in the cerebrospinal fluid (CSF). Our bioinformatics analysis suggests that these miRNAs modulate pathways associated with senescence and cellular stress response, targeting crucial genes such as *Cdkn2a*, *Rps27*, and *Txnip*. The oligodendrocyte lineage appears to be the most responsive to age-dependent loss of exosomal miRNA, leading to significant derepression of several miRNA target genes. Furthermore, heterochronic parabiosis can reverse age-related upregulation of specific miRNA-targeted genes, predominantly in brain endothelial cells, including senescence promoting genes such as *Cdkn1a* and *Btg2*. Our findings support the presence of an anti-senescence mechanism triggered by the endocrine secretion of htNSC-derived exosomal miRNAs, which is associated with a youthful transcriptional signature.

## 1. Introduction

The progressive decline in physiological functions as individuals age has traditionally been attributed to the accumulation of stochastic damage in macromolecules, a concept that aligns with the free radical theory of aging [1]. Additionally, aging can be conceptualized as a diminishing capacity of protective, reparative, and regenerative mechanisms that normally maintain youthfulness. This notion of a “longevity program”, orchestrated by endocrine factors, is evidenced by the rejuvenation effects observed in aged organisms undergoing heterochronic parabiosis with a younger counterpart [2].

Circulating exosome-packaged miRNAs are emerging as promising candidates capable of exerting systemic effects within a potential anti-aging program. These miRNAs are small, noncoding RNAs that function within ribonucleoprotein silencing complexes, pairing with target mRNAs to induce their degradation or translational repression [3]. Their ability to regulate numerous genes simultaneously, coupled with their capacity for intercellular communication via various carriers found in bodily fluids, including exosomes (nanosized vesicles released by cells), makes miRNAs well-suited for orchestrating cellular functional programs at the organismal level [4].

The paramount cell program associated with aging is cellular senescence, a response activated by various types of damage including genotoxic, mitochondrial, or oxidative injuries [5]. As organisms age, the accumulation of senescent cells intensifies, leading to the secretion of multiple extracellular mediators known as the senescence-associated secretory phenotype (SASP). These mediators induce the senescence of nearby cells, a process referred to as secondary or paracrine senescence [6]. Cellular senescence is characterized by a stable proliferative arrest mediated by the activation of the tumor suppressors TP53 and CDKN2A, and their downstream effectors, CDKN1A and RB1 family proteins [7]. Even in a primarily post-mitotic tissue like the brain, accumulation of senescent cells with aging had been observed [8].

Recently, it has been discovered that adult NSCs localizing in the mediobasal hypothalamic parenchyma, and the third-ventricle wall of the hypothalamus are almost completely lost with aging, correlating with age-associated physiological decline. The loss of htNSC is associated with a stable and chronic background inflammation associated with the early stages of aging or metabolic syndrome [9]. Cellular senescence and inflammation are intertwined, as several components of the SASP are pro-inflammatory molecules. At the organismal level, immunosenescence generates an age-related chronic proinflammatory process, termed inflammaging [10]. Interestingly, senescent cell clearance reduces age-associated microglial activation, neuroinflammation, and immune cell infiltration, thereby mitigating cognitive decline [11,12]. Moreover, reducing senescent microglia reduced brain inflammation and improved cognition in a mice model of Alzheimer’s disease [13]. These findings underscore the importance of the accumulation of senescent cells in initiating neurodegenerative processes by promoting inflammation [14].

htNSCs actively secrete exosomal miRNAs, and exosomes derived from htNSCs can attenuate age-related declines in cognitive abilities and muscle function when administered to mid-aged mice [15]. However, the specific transcriptional programs regulated by these miRNAs remain unstudied. We hypothesize that these miRNAs may modulate anti-senescence pathways, playing a role in the cell-specific transcriptional changes observed during brain aging.

In support of this assumption, recent observations have shown that extracellular vesicle (EV)-packaged miRNAs secreted by embryonic stem cells (ESCs) can rejuvenate the transcriptome profile of aged fibroblasts in vitro. Furthermore, intraperitoneal injection of ESC-EVs to mid-aged mice reduces senescent cell number in several organs, including the spleen, kidney, and liver, and decreases the expression of age-related genes such as *p16*, *p21*, and *p53* [16]. Additionally, intravenous injection of purified small EVs from young mice (2 months) into aged mice (20 months) significantly extends the median lifespan, reduces the expression of senescent makers in several tissues, and promotes miRNA-mediated improvement in mitochondrial function [17].

Here, using bioinformatic tools, we identified a putative anti-senescence gene regulatory program triggered by exosomal miRNAs derived from htNSCs, which correlates with the transcriptional changes observed in the aged mice brain. Additionally, we identified that heterochronic parabiosis can reverse the age-related increased expression of specific genes targeted by these miRNAs, particularly in brain endothelial cells, further supporting the theory of an endocrine control of the aging process.

## 2. Results and Discussion

### 2.1. Pathways Associated with htNSC-Derived miRNA Target Genes

A total of 19 exosomal miRNAs, preferentially secreted by htNSCs, were found significantly reduced in the CSF of middle-aged mice (16 months) by Zhang et al. [15]. Due to limited experimental knowledge about miRNA target genes in mice, we utilized information about their human orthologs. This approach is supported by the widespread conservation of miRNA–mRNA pairs in mammals, particularly in widely conserved miRNA families [18]. We successfully mapped 11 murine miRNAs to their corresponding human orthologs in the miRBase [19], revealing high sequence identity and strict conservation of their 7-mer seeds (Supplementary Table S1). MiR-483-5p was excluded from further analysis due to poor family conservation.

**Figure 1:**
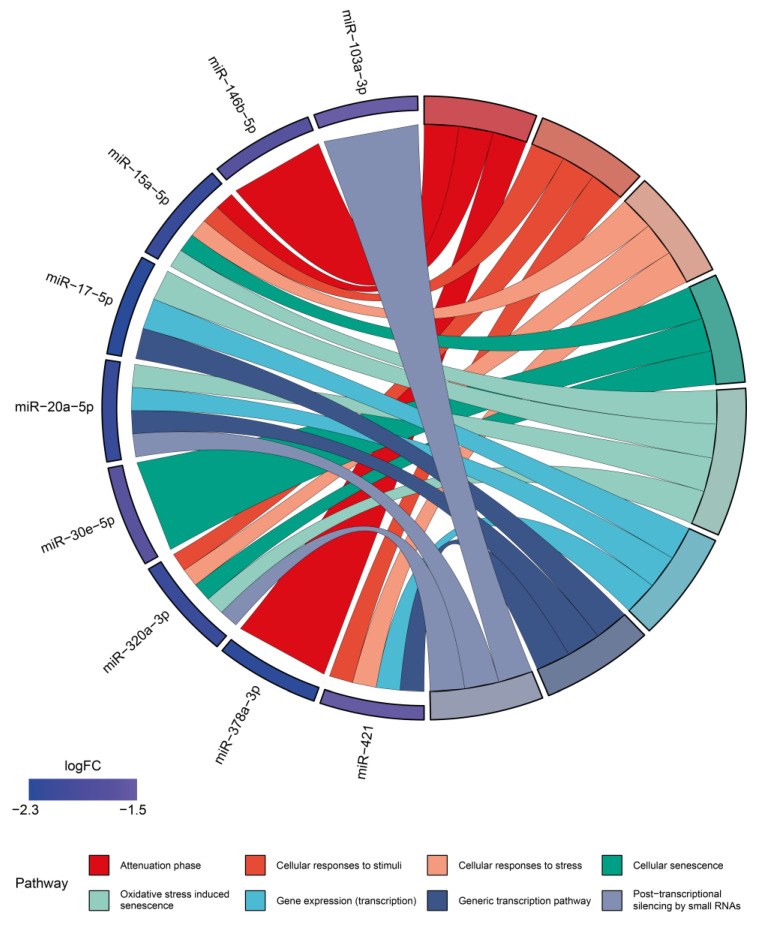
Shared pathways enriched in htNSC-derived miRNA gene targets suggest a transcriptional anti-aging program operating in young animals. Chord diagram showing Reactome pathways shared by ≥3 htNSCs miRNA-targeted genes. Logarithmic fold change (logFC) in miRNA CSF levels with aging was reported in [5]. Figure was made with SRPlot (http://www.bioinformatics.com.cn/SRplot (accessed on 30 November 2023)).

Therefore, experimentally validated miRNA–target interactions extracted from miRTarBase 9.0 [20] were analyzed in Reactome release 78 [21] to identify pathways enriched in the genes targeted by each miRNA. The ten most enriched pathways in genes targeted by each miRNA are shown in Supplementary Figure S1. Except for miR-30a-5p, the genes targeted by all the other miRNAs share several enriched pathways (Figure 1), which can be grouped into two hierarchical classes.

On one hand, we observed enrichment in the Cellular responses to stimuli pathway (R-HSA-89533897) and its related sub-pathways, including Cellular responses to stress (R-HSA-2262752), Attenuation phase (R-HSA-33715568), Cellular senescence (R-HSA-2559583), and Oxidative stress-induced senescence (HSA-2559580). On the other hand, there was enrichment in the Gene expression (transcription) pathway (R-HSA-74160) and its sub-pathways, such as the Generic transcription pathway (R-HSA-212436) and Post-transcriptional silencing by small RNAs (R-HSA-426496).

Considering that miRNAs act through post-transcriptional suppression of specific gene targets, our analysis suggests that they can control transcriptional programs, antagonizing pathways associated with cellular senescence, particularly downstream of oxidative stress. The loss of this inhibitory control could explain the acceleration of aging occurring when the htNSC-derived exosomal miRNA levels are diminished [15].

Our results are consistent with numerous previous studies showing that some of the htNSC-derived miRNAs exert anti-senescence and/or pro-longevity functions. These include miR-17-5p, a miRNA downregulated during aging in different model systems [22]. Interestingly, miR-17 overexpression in mice decreases cell senescence and extends lifespan [23], constituting the first experimentally validated “longevi-miR” [24]. On the other hand, miR-146b-5p reduces IL-6 and IL-8 secretion (two components of SASP) by senescent fibroblasts, restraining the inflammatory response [25]. This effect is replicated in human trabecular meshwork cells, concomitantly with a reduction in ROS production and senescence markers [26]. Similarly, mir-378a-5p counteracts fibroblast senescence, reducing the levels of p16^INK4a^, a tumor suppressor that has a central role in the induction of cell senescence [27]. Finally, miR-421 also suppresses senescence through the downregulation of ATM expression in prostate cancer cells [28].

### 2.2. Brain Cell-Specific Transcriptional Changes Putatively Associated with Age-Dependent Decline in htNSC-Derived miRNAs

**Figure 2:**
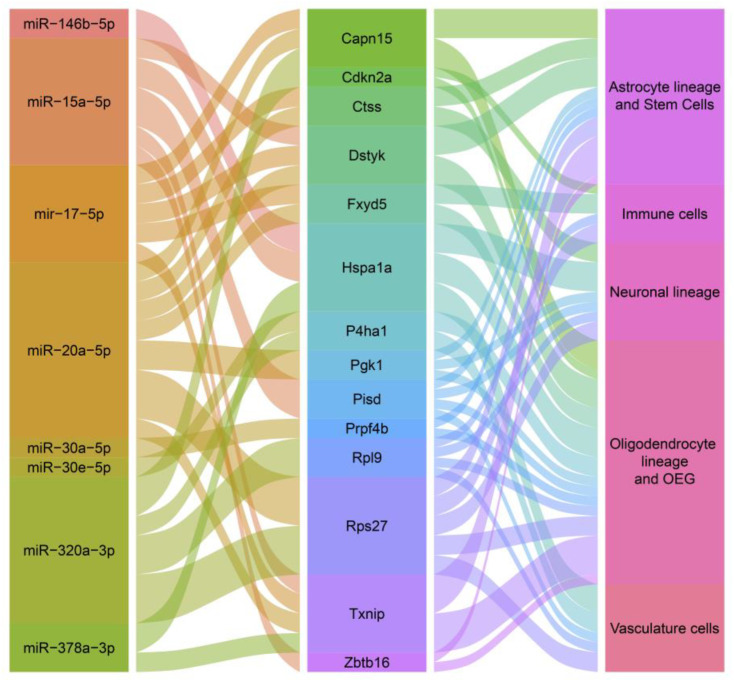
Age-upregulated htNSC-derived miRNA target genes across the main brain cell classes. Alluvial plot networking htNSC-secreted miRNAs with their target genes upregulated with aging in ≥2 brain cell classes. Statistically significantly upregulated genes determined by single-cell RNA-seq (TPM-based FC ≥ 1.5 and adjusted *p*-value < 0.05) were extracted from [16].

Considering that mRNA destabilization explains most (66 to 90%) of the miRNA-mediated repression [3], we hypothesize that changes in mRNA levels during brain aging may in part reflect transcriptional programs controlled by miRNAs. Accordingly, we mined a comprehensive dataset of single-cell transcriptomics of the aged mouse brain [29], in search of htNSC-derived miRNA gene targets. We focused on those genes that show a significant increase in mRNA levels during aging, consistent with the age-dependent decline in miRNA levels in CSF. Genes upregulated by aging in two or more brain cell groups with their respective regulating miRNAs are shown in Figure 2. Age-dependent loss of miRNA-mediated downregulation of several of these genes can be associated with accelerated aging phenotypes. For example, age-dependent upregulation of thioredoxin-interacting protein (TXNIP), a target of miR-15a-5p, miR-17-5p, miR-20-5p, and miR-378a-3p, can result in reduced thioredoxin-1 activity and decreased resistance to oxidative stress. Interestingly, siRNA-mediated depletion of TXNIP in *Drosophila melanogaster* significantly extended median lifespan [30], highlighting it as an important target of the anti-aging program triggered by htNSC-derived miRNAs. Additionally, supporting this notion, increased TXNIP expression in the aged mouse brain triggers inflammasome upregulation, and pharmacological reduction in TXNIP levels has rejuvenating effects, improving cognitive and sensorimotor abilities [31].

Another interesting target gene of htNSC-derived miRNAs that shows upregulation in the aging brain is *Rps27*, which encodes a zinc finger-containing protein component of the 40S ribosomal subunit. The knockdown of *rps-27* (the ortholog of mammalian *Rps27*) in *Caenorhabditis elegans* resulted in 44–50% increased lifespan [32], highlighting it as another relevant target of the anti-aging program.

**Figure 3:**
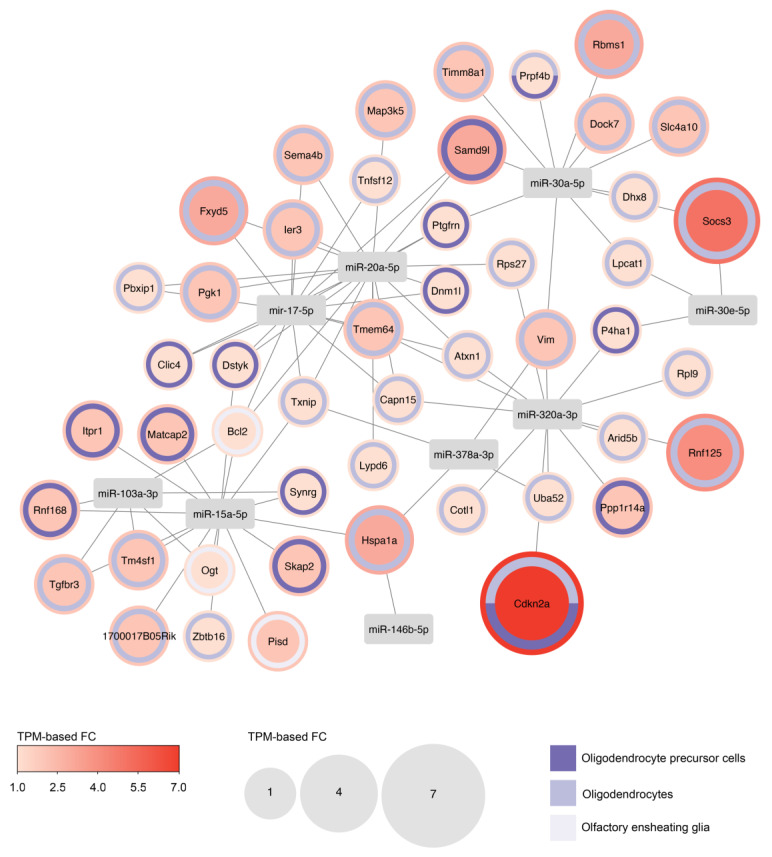
Interaction network between htNSC-derived miRNAs and their age-upregulated target genes in oligodendrocyte lineage. Regulatory gene network between miRNAs and age-upregulated genes in the oligodendrocyte precursor cells, mature oligodendrocytes, and olfactory ensheathing glia. Statistically significantly upregulated genes determined by single-cell RNA-seq (TPM-based FC ≥ 1.5 and adjusted *p*-value < 0.05) were extracted from [16]. For genes upregulated in more than one cell type, node size and color represent the greatest change observed.

To gain further insight into the potential consequences on the transcriptome of the loss of these miRNAs during aging, we constructed cell-specific gene networks, linking age-upregulated genes with their respective regulating miRNAs (Figure 3 and Supplementary Figure S2). Among the cell classes analyzed, the lineage of oligodendrocytes and the olfactory ensheathing glia undergo the most significant remodeling of the transcriptome. This is evident in both the number of upregulated genes and the magnitude of the age-dependent changes observed (Figure 3).

Interestingly, the aging process is associated with a decrease in the differentiation potential of oligodendrocyte progenitor cell (OPC), leading to reduced production of oligodendrocytes and a failure in remyelination [33]. Among the many genes upregulated with age, the increased expression of *Cdkn2a*, a target of miR-320a-3p, is observed in both OPC and mature oligodendrocytes (Figure 3). The *Cdkn2a* locus encodes the cell cycle inhibitors p16^INK4a^ and p19^ARF^, which are highly expressed in senescent cells and play a crucial role in establishing cellular senescence [34]. In the aged rat brain, OPCs exhibit classical hallmarks of aging, including an 8-fold higher mRNA of *Cdkn2a*, which may be mitigated by interventions such as intermittent fasting or metformin treatment [35]. Notably, direct infusion of young CSF into aged brains primarily impacts on the transcriptome of hippocampal oligodendrocytes, promoting OPC proliferation and differentiation, and improving memory function [36]. We hypothesize that exosomal miRNAs secreted by htNSCs present in young CSF could be partially responsible for these rejuvenating changes. Thus, our analysis suggests that the oligodendrocyte lineage is a crucial target of the anti-senescence program mediated by htNSC-derived exosomal miRNAs, which is compromised with aging, leading to impaired oligodendrogenesis.

### 2.3. Heterochronic Parabiosis Antagonizes Age-Associated Upregulation of Several Target Genes of htNSC-Derived miRNAs

Due to their location in hypothalamic third-ventricle wall and the mediobasal hypothalamic parenchyma, secretory products of htNSCs can reach both the CSF and the bloodstream. This occurs through the fenestrated capillaries of the median eminence, where the blood–brain barrier is interrupted [37]. The presence of htNSC-secreted exosomal miRNAs in the bloodstream could partly explain their endocrine effects. This leads us to speculate that they could mediate the rejuvenating effects observed in heterochronic parabiosis, a surgical procedure that connects the circulatory systems of young and old mice. To evaluate this hypothesis, we analyzed a dataset of transcriptome-wide changes across 31 major brain cell types after heterochronic parabiosis [38]. We focused on genes targeted by htNSC-derived miRNAs that show a reversal in their age-associated upregulation.

**Figure 4:**
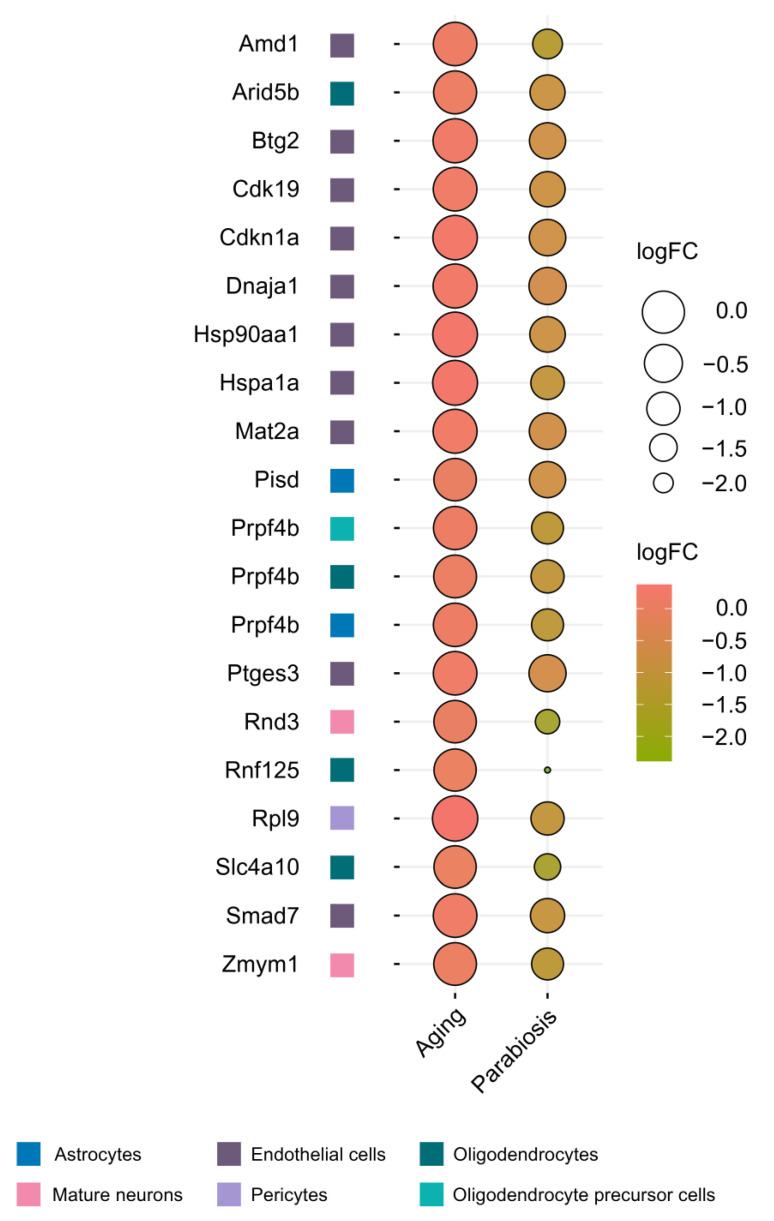
Heterochronic parabiosis reverse age-dependent upregulation of several target genes of htNSC-secreted exosomal miRNAs. Balloon plot showing the reversal of age-associated upregulation of htNSC-derived miRNA target genes after 4–5 weeks of heterochronic parabiosis. Logarithmic-transformed FCs were downloaded from [16] (aging-associated changes) and [20] (heterochronic parabiosis-associated changes). We used a Benjamini–Hochberg-adjusted *p*-value < 0.05 thresholding to determine statistically significant differential expression.

Our analysis reveals that the most significant changes in miRNA-targeted genes occur in endothelial cells (Figure 4), remarkably, the cell type directly influenced by exosomes found in young blood. This finding aligns with previous studies, indicating that brain capillary endothelial cells are highly sensitive to age-related signals from the circulatory system, as demonstrated by the rejuvenation on their gene expression profiles following infusions of young plasma [39].

Recent findings suggest that approximately 10% of cerebromicrovascular endothelial cells undergo cellular senescence in the 28-month-old mouse brain [40], a phenomenon associated with compromised integrity of the blood–brain barrier (BBB) [41].

Consistent with a putative anti-senescence program triggered by htNSC miRNAs, our bioinformatic analysis identified two senescence-associated genes, *Btg2* and *Cdkn1a* (both targeted by miR-15a-5p, miR-17-5p, and miR-20a-5p), whose age-dependent upregulation is reversed by heterochronic parabiosis. BTG2 is an antiproliferative protein that operates downstream of p53 to induce cell growth arrest [42]. Depletion of BTG2 in human fibroblasts extends cellular lifespan, while ectopic BTG2 induces senescence [43]. BTG2 also plays a role in promoting senescence in muscle stem cell and cardiomyocytes [44,45], indicating its involvement in senescence signaling across various cell types. On the other hand, the *Cdkn1a* gene encodes p21^Cip/Waf1^, which is induced during cellular senescence [46] and controls a telomere dysfunction-mediated checkpoint that can limit longevity in mice [47].

Further experiments are required to evaluate the specific contribution of each of these miRNAs to the transcriptional rejuvenation observed across different brain cell types, as well as their potential role in preventing cellular senescence.

### 2.4. Widespread Transcriptional Changes in Aged Hypothalamus Are Putatively Associated with a Decline in htNSC-Derived miRNAs

Recently, it has been observed that reduced expression of specific miRNAs during aging is reflected at the transcriptome level by a loss or decline in the repression of gene expression of their putative target genes [48]. Inspired by this observation, we delved into the transcriptional changes in the aged hypothalamus to investigate the local effects of the decrease in htNSC-derived exosomal miRNAs. To achieve this objective, we analyzed the differentially expressed genes in the hypothalamus between young (2 months) and aged (19–24 months) female mice across 11 major cell types [49]. Our focus was on identifying widespread changes across these principal hypothalamus cell types, to avoid potential cell-specific alterations inconsistent with a paracrine regulation of gene expression by exosomal miRNAs.

**Figure 5:**
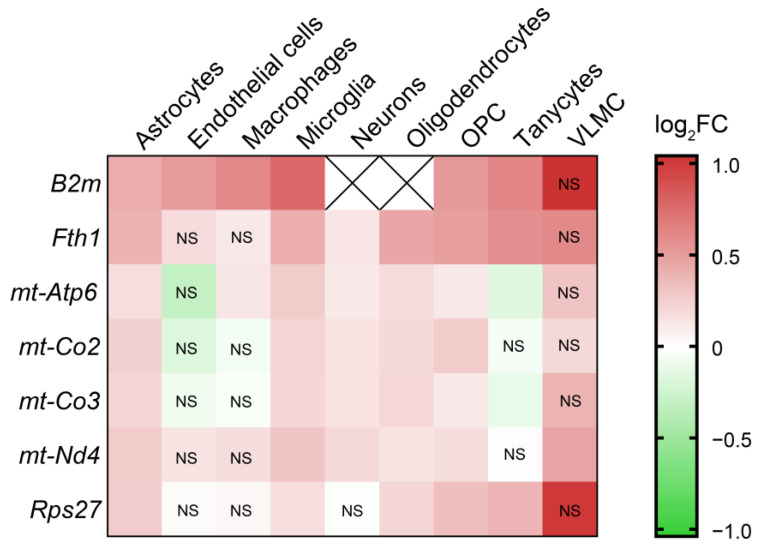
htNSC-derived miRNA target genes are widely upregulated in aged hypothalamus. Heatmap highlighting differential expression of htNSC-derived miRNA target genes between young and old female hypothalamus-specific cell types. Color scale indicates the logarithmic-transformed FC. NS = not significant.

Seven target genes of htNSC-derived miRNAs consistently show upregulation in at least five cell types in the aged hypothalamus (Figure 5). These genes include *Rps27* (also found upregulated in the whole brain dataset, as previously discussed), *B2m*, *Fth1*, three mitochondrially encoded subunits of the electron transport chain complexes (*mt*-*Co2*, *mt-Co3* and *mt-Nd4*), and one subunit of the ATP synthase complex (*mt-Atp6*).

*B2m* encodes β2-microglobulin (B2M), a component of major histocompatibility complex class 1 (MHC I) molecules. Interestingly, B2M is upregulated and accumulates on the surface of senescent cells [50], constituting a bona fide senescent marker [51]. Moreover, the absence of endogenous B2M expression abolishes age-related cognitive decline and enhances neurogenesis in aged mice [52], suggesting a role as a pro-aging factor, potentially suppressed by htNSC-derived miRNAs in young animals.

On the other hand, *Fth1* encodes the ferritin heavy chain (H-ferritin), whose ferroxidase activity converts Fe^2+^ to Fe^3+^, allowing iron atoms to be safely stored in the ferritin complex. Interestingly, an increase in iron levels and upregulation of *Fth1* and *Ftl* (encoding the ferritin light chain) is observed early upon senescence induction by cell damage agents [53]. Concordantly, senescent cells increase H-ferritin expression by 10-fold, concurrently with an increase in intracellular iron levels [54]. Therefore, the suppression of the senescent phenotype through the regulation of iron accumulation by htNSC-derived miRNAs emerges as a promising mechanism worthy of further exploration.

Furthermore, the identification of mitochondrial-located mRNAs as putative targets of miRNAs is not surprising, since the mitochondrial localization of many miRNAs has been described [55] and the gene-silencing machinery by RNA interference is active inside mitochondria [56], regulating mitochondrial RNA expression [53]. Increased mitochondrial biogenesis and impaired turnover lead to the accumulation of mitochondria in senescent cells, accompanied by extensive metabolic rewiring [57,58]. Moreover, a broad number of senescence-associated changes are dependent on mitochondria, and a reduction in mitochondrial content prevents senescence in the aging mouse liver [58]. Whether htNSC-secreted miRNAs antagonize the increase in mitochondrial mass as part of their anti-senescence program may be the subject of future studies.

## 3. Materials and Methods

### 3.1. htNSC-Secreted Exosomal miRNA Target Genes

The lists of experimentally validated target genes of miRNAs were obtained from miRTarBase 9.0 [20]. This includes target genes supported by “strong” experimental evidence such as reporter assays, Western blot and/or qPCR, or “less strong” evidence derived from high-throughput techniques like Photoactivatable Ribonucleoside-Enhanced (PAR)-Crosslinking and Immunoprecipitation (CLIP), high throughput sequencing (HITS)-CLIP or crosslinking ligation and sequencing of hybrids (CLASH). Human genes were then mapped to their respective mouse orthologs using the HGNC Comparison of Orthology Predictions (HCOP) [59].

### 3.2. miRNA Target Gene Pathway Analysis

Pathway enrichment analysis of miRNA target genes was performed in Reactome release 78 [21] using human Uniprot identifiers. Over-representation was assessed through a statistical test based on the hypergeometric distribution, generating a probability score that was then corrected for false discovery rate (FDR) using the Benjamini–Hochberg method.

### 3.3. Single-Cell RNA Sequencing of Brain Cells during Aging and Heterochronic Parabiosis

Differentially expressed genes (DEGs) between young male mice (2–3 months of age) and old male mice (21–22 months) across 25 brain cell types were obtained from Ximerakis et al. [29]. Upregulated genes were defined as those with a fold change (based on the transcripts per kilobase million (TPM) values) from young to old ≥ 1.5 and an FDR-adjusted *p*-value < 0.05. Conversely, DEGs between heterochronic parabionts (3–4-month-old mice joined with 20–22-month-old counterparts) and their isochronic parabionts across 31 major brain cell types were extracted from Ximerakis et al. [38]. Downregulated genes were determined by a logarithmic-transformed fold change (logFC) < 0 and an FDR-adjusted *p*-value < 0.05.

DEGs between young (3 months) and aged (19–24 months) female mouse hypothalamus was downloaded from Hajdarovic et al. [49]. Genes were considered significantly upregulated if the adjusted *p*-value was <0.05 and the log_2_FC was >0.1.

### 3.4. Graphics

Graphics were generated using SRplot web server (http://www.bioinformatics.com.cn/SRplot (accessed on 30 November 2023)) [60], GraphPad Prism 10, and Cytoscape 3.10.1 [61] with yFiles layout algorithms and Legend creator add-on.

## 4. Conclusions

Age-associated neuroinflammation leads to almost complete loss of hypothalamus-residing NSCs in older mice. This event triggers the decline in htNSC secretory products such as exosomal miRNAs, which contributed to maintaining physiological fitness in young animals [15,62]. To complete the puzzle, we showed that these miRNAs control transcriptional programs associated with cellular responses to stress, particularly cellular senescence. Analysis of transcriptional signatures associated with aging and heterochronic parabiosis, across the main brain cell types and especially in the aged hypothalamus, revealed putative key genes of the anti-senescence program controlled by these miRNAs. Furthermore, we identify the oligodendrocyte lineage and endothelial cells as the presumptive main targets of these hypothalamic-derived exosomal miRNAs.

**Figure 6:**
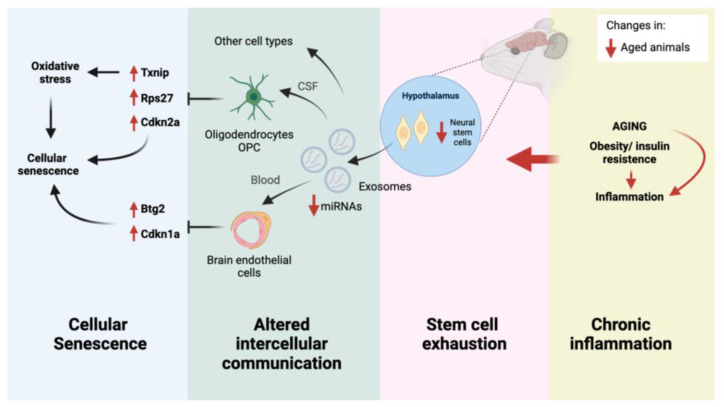
Integrative model of the anti-senescence program triggered by htNSC-derived miRNAs with the hallmarks of aging. Aging-dependent inflammation causes htNSC loss and diminished exosomal miRNA secretion. Subsequent loss of decline in the repression of key target genes promotes brain aging through cellular senescence and oxidative stress.

The current molecular model of aging proposes twelve hallmarks, grouped into three categories: primary (causes of damage), antagonistic (responses to damage), and integrative (responsible for age-associated functional decline) [63]. Our work merges three integrative hallmarks, chronic inflammation, stem cell exhaustion, and altered intercellular communication with an antagonistic hallmark, cellular senescence (Figure 6).

A decade of studies has established that the hypothalamus functions as a central control center of systemic aging through various intercellular communication pathways [64,65]. Particularly important is the positive feedback loop between white adipose tissue (WAT) and the hypothalamus, which promote neural activity and lipolysis, respectively, delaying aging and extending lifespan in mice [66,67]. Interestingly, depletion and impaired neuronal differentiation of adult htNSCs can be triggered by dietary obesity and glucose intolerance [68], supporting the connection between metabolic disorders and accelerated aging [69]. Preserving htNSC activity including exosomal miRNA secretion can keep the anti-aging program active in older individuals, supporting healthy aging and promoting longevity.

Our analysis has several limitations. As a purely bioinformatic study, the correlations obtained here require experimental validation, especially to find causal relationships between the age-dependent fall in CSF miRNA levels and a loss of or decline in gene repression of its target genes. Intracerebroventricular injection of young htNSC-derived exosomes coupled to single-cell transcriptomics of brain cells could help to establish such causal relationships. In addition, the use of antagomiRs, modified oligonucleotides that produce a potent and specific inhibition of miRNAs in vivo, could elucidate hierarchical or synergistic effects of the htNSC-secreted miRNAs in the anti-senescence transcriptional program.

## Data Availability

The data that support the findings of this study are available from the corresponding author upon request.

## Supplementary Materials

The following supporting information can be downloaded at: https://www.mdpi.com/article/10.3390/ijms25105467/s1.
