# Supplementary material for: A Brain Anti-Senescence Transcriptional Program Triggered by Hypothalamic-Derived Exosomal microRNAs
Source: Int J Mol Sci. 2024 May 17;25(10):5467. doi: 10.3390/ijms25105467 (PMC11122052; doi:10.3390/ijms25105467)
Supplement: Supplementary file 1 [file ijms-25-05467-s001.zip › Supplementary figure 2 (1).pdf]

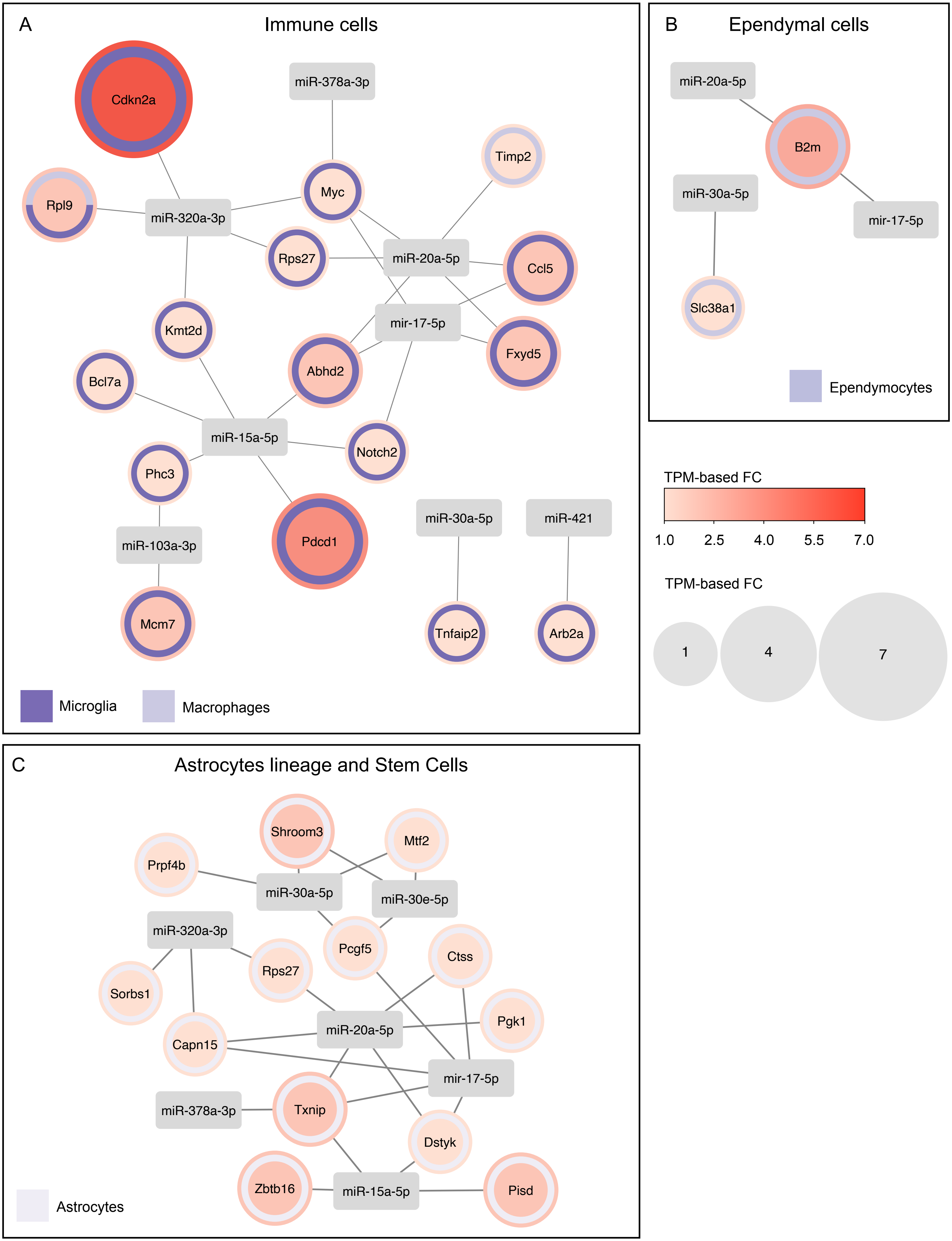
A
Immune cells
Cdkn2a
miR-378a-3p
Timp2
Myc
Rpl9
miR-320a-3p
Rps27
miR-20a-5p
Ccl5
mir-17-5p
Kmt2d
Fxyd5
Abhd2
Bcl7a
miR-15a-5p
Notch2
Phc3
Pdcd1
miR-30a-5p
miR-421
miR-103a-3p
Mcm7
Tnfaip2
Arb2a
Microglia
Macrophages
B
Ependymal cells
miR-20a-5p
B2m
miR-30a-5p
mir-17-5p
Slc38a1
Ependymocytes
TPM-based FC
1.0
2.5
4.0
5.5
7.0
TPM-based FC
1
4
7
C
Astrocytes lineage and Stem Cells
Shroom3
Mtf2
Prpf4b
miR-30a-5p
miR-30e-5p
miR-320a-3p
Pcgf5
Ctss
Rps27
Sorbs1
Pgk1
miR-20a-5p
Capn15
mir-17-5p
miR-378a-3p
Txnip
Dstyk
Zbtb16
miR-15a-5p
Pisd
Astrocytes

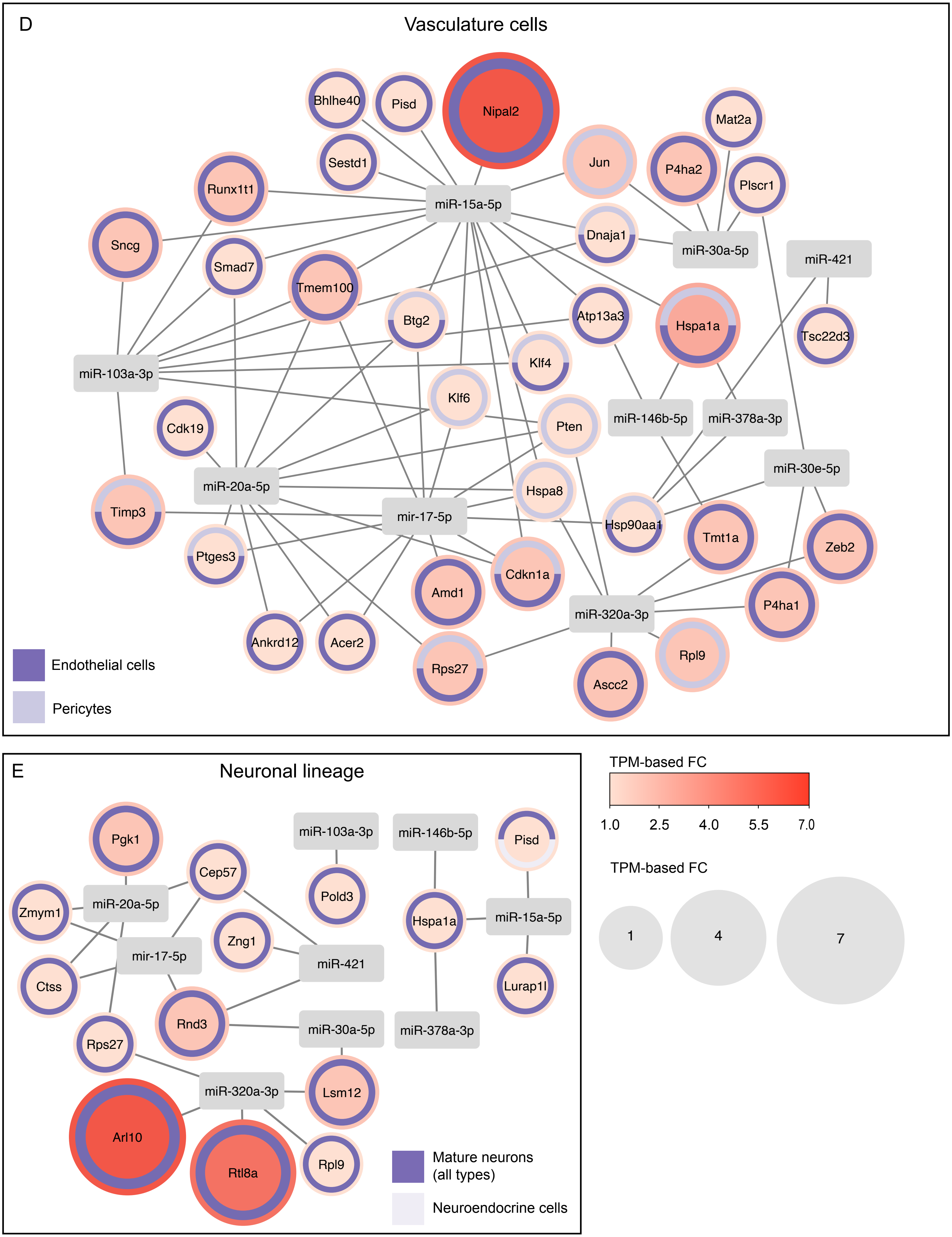

D
Vasculature cells
Nipal2
Bhlhe40
Pisd
Sestd1
Jun
P4ha2
Mat2a
Plscr1
Runx1t1
miR-15a-5p
Sncg
Smad7
Dnaja1
miR-30a-5p
miR-421
Tmem100
Btg2
Atp13a3
Hspa1a
Tsc22d3
miR-103a-3p
Klf4
Klf6
Pten
miR-146b-5p
miR-378a-3p
Cdk19
miR-30e-5p
miR-20a-5p
Hspa8
Timp3
mir-17-5p
Hsp90aa1
Tmt1a
Zeb2
Ptges3
Cdkn1a
Amd1
miR-320a-3p
P4ha1
Ankrd12
Acer2
Rps27
Rpl9
Ascc2
Endothelial cells
Pericytes
E
Neuronal lineage
Pgk1
Cep57
miR-103a-3p
miR-146b-5p
Pisd
Zmym1
miR-20a-5p
Pold3
Hspa1a
miR-15a-5p
Zng1
mir-17-5p
miR-421
Ctss
Lurap1l
Rnd3
Rps27
miR-30a-5p
miR-378a-3p
miR-320a-3p
Lsm12
Arl10
Rtl8a
Rpl9
Mature neurons (all types)
Neuroendocrine cells
TPM-based FC
1.0
2.5
4.0
5.5
7.0
TPM-based FC
1
4
7
