# Supplementary figures and images for: A Brain Anti-Senescence Transcriptional Program Triggered by Hypothalamic-Derived Exosomal microRNAs
Source: Int J Mol Sci. 2024 May 17;25(10):5467. doi: 10.3390/ijms25105467 (PMC11122052; doi:10.3390/ijms25105467)

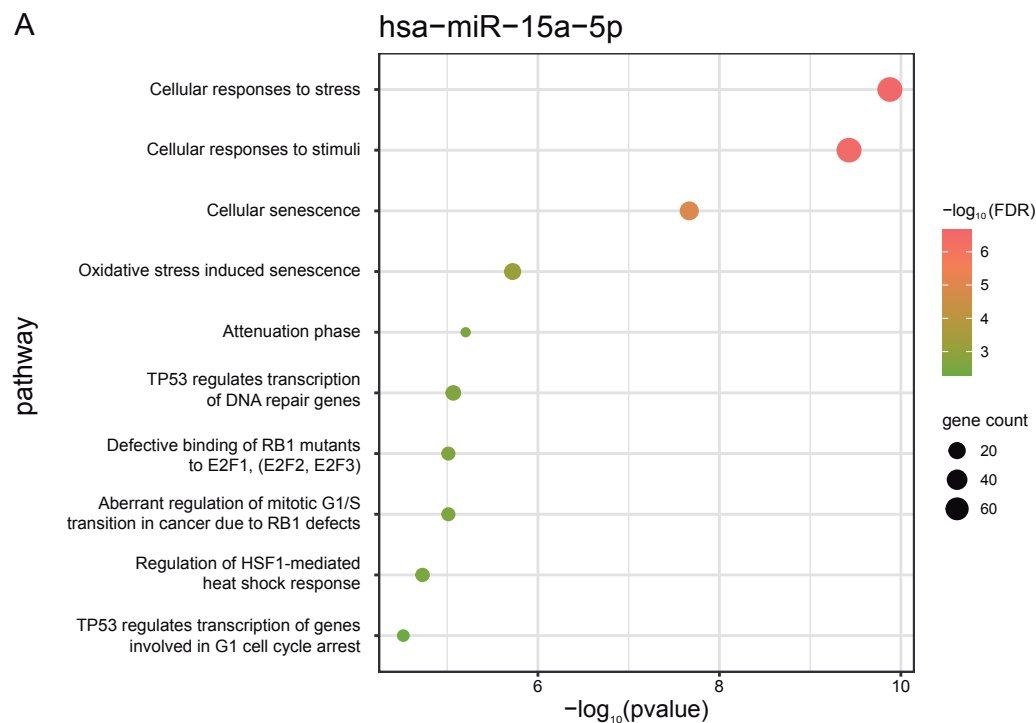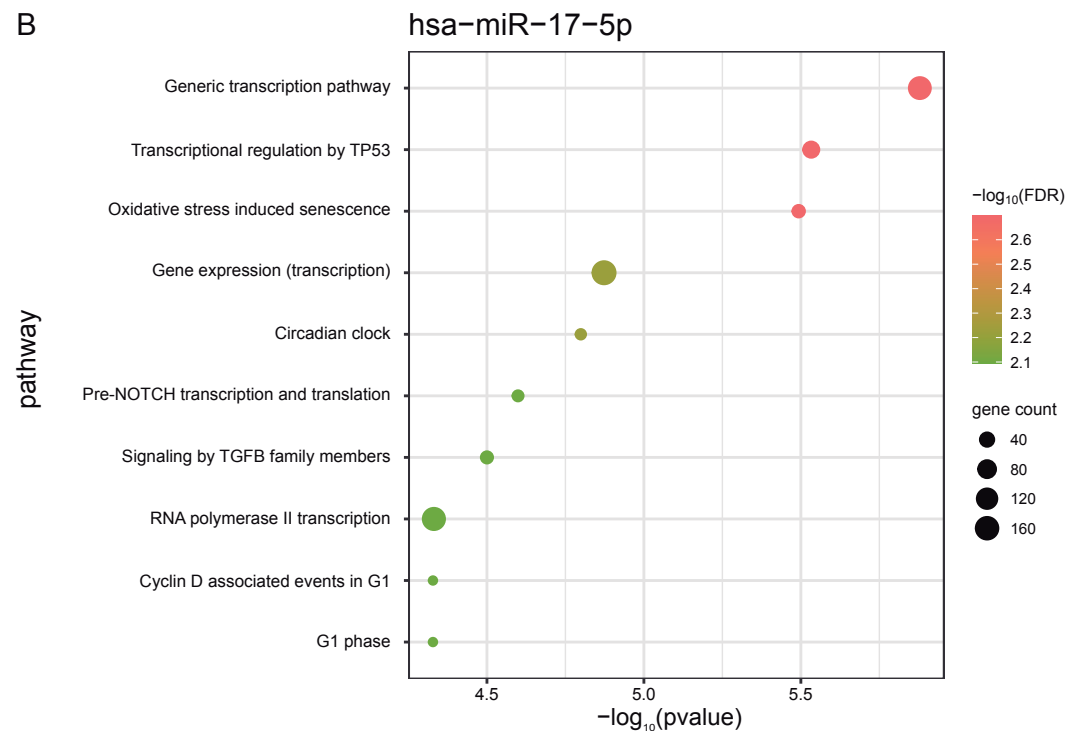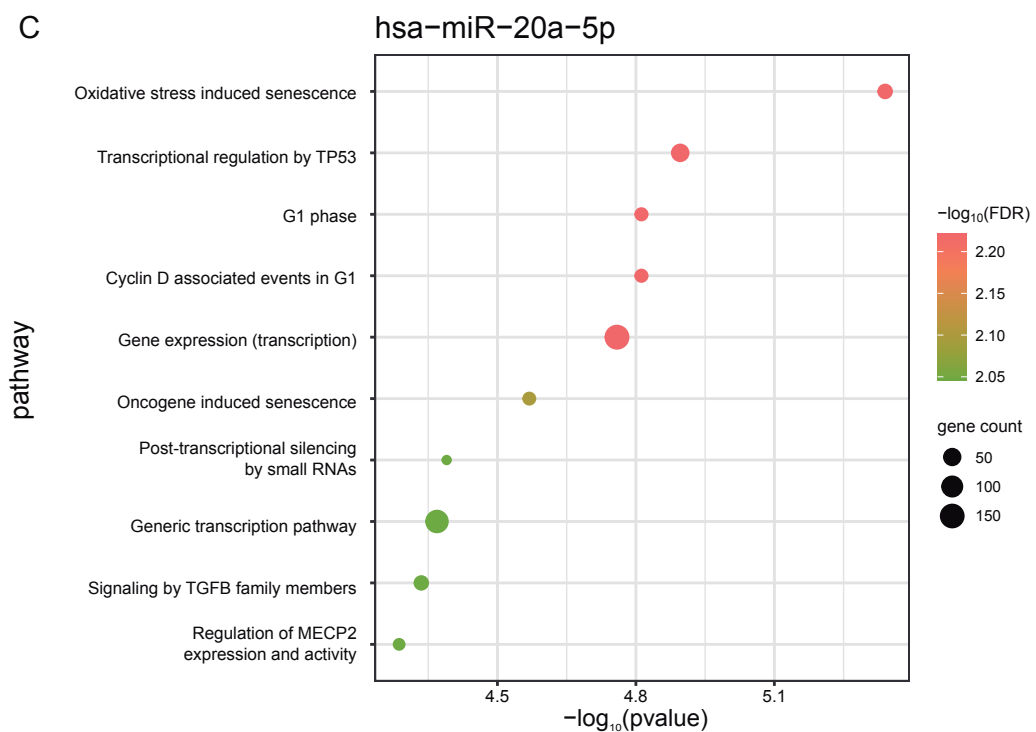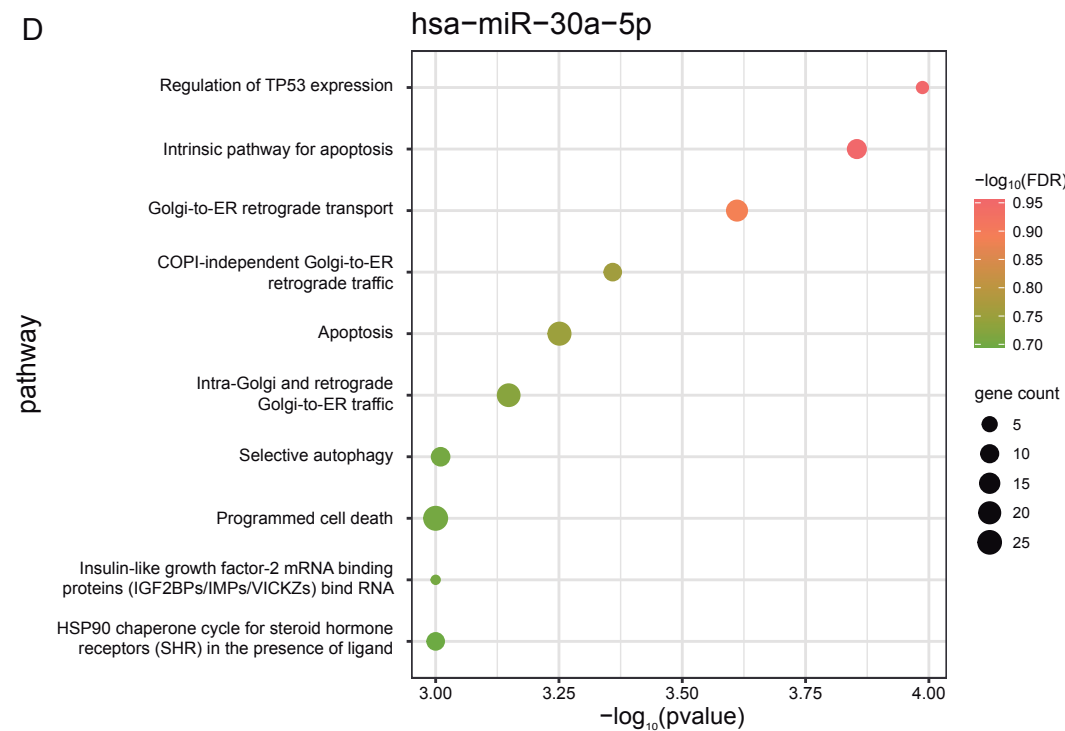

E

hsa-miR-30e-5p

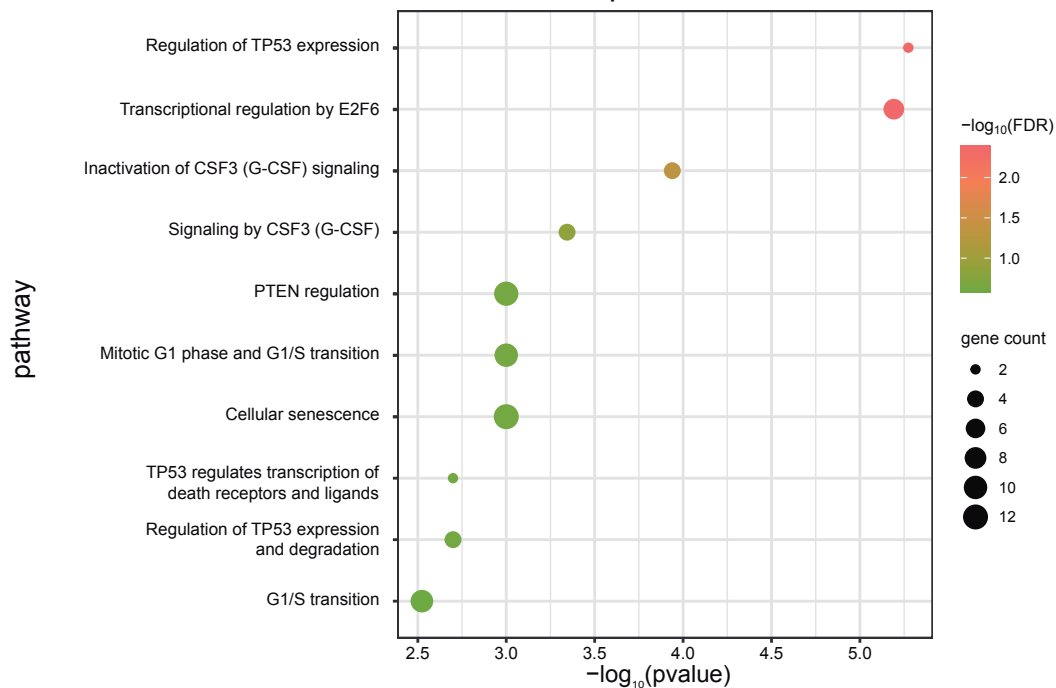

F

hsa-miR-103a-3p

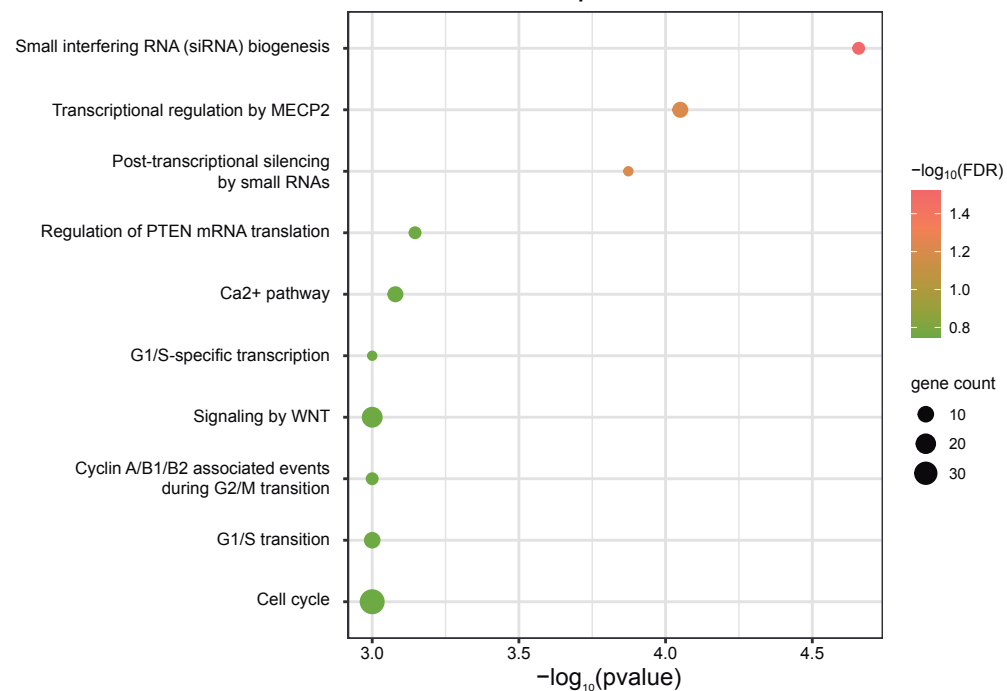

G

hsa-miR-146b-5p

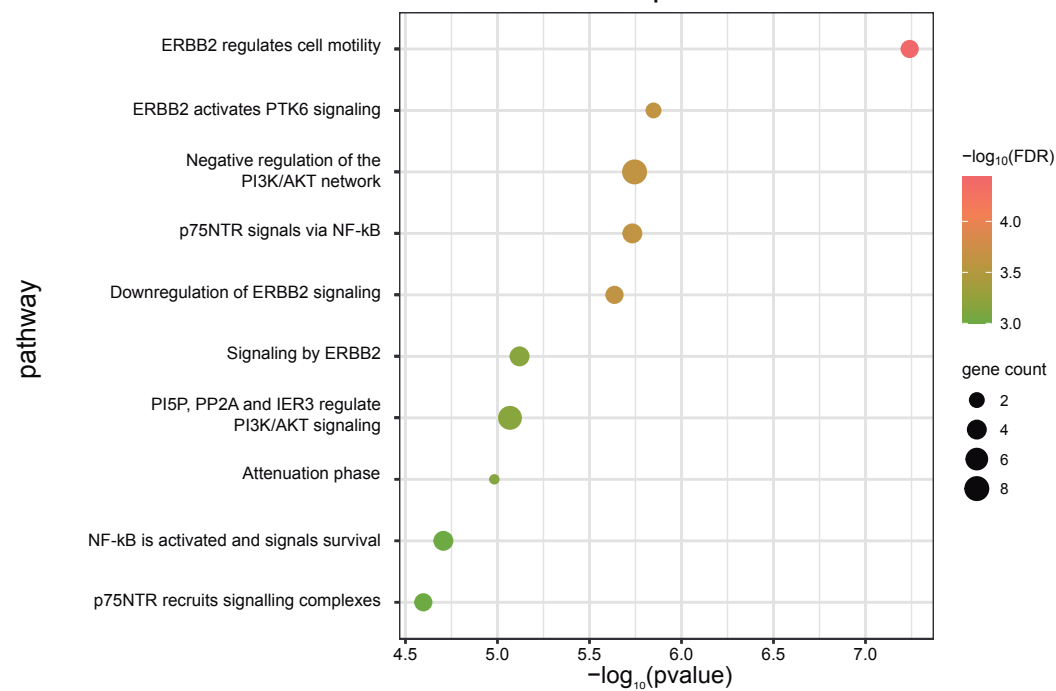

H

hsa-miR-320a-3p

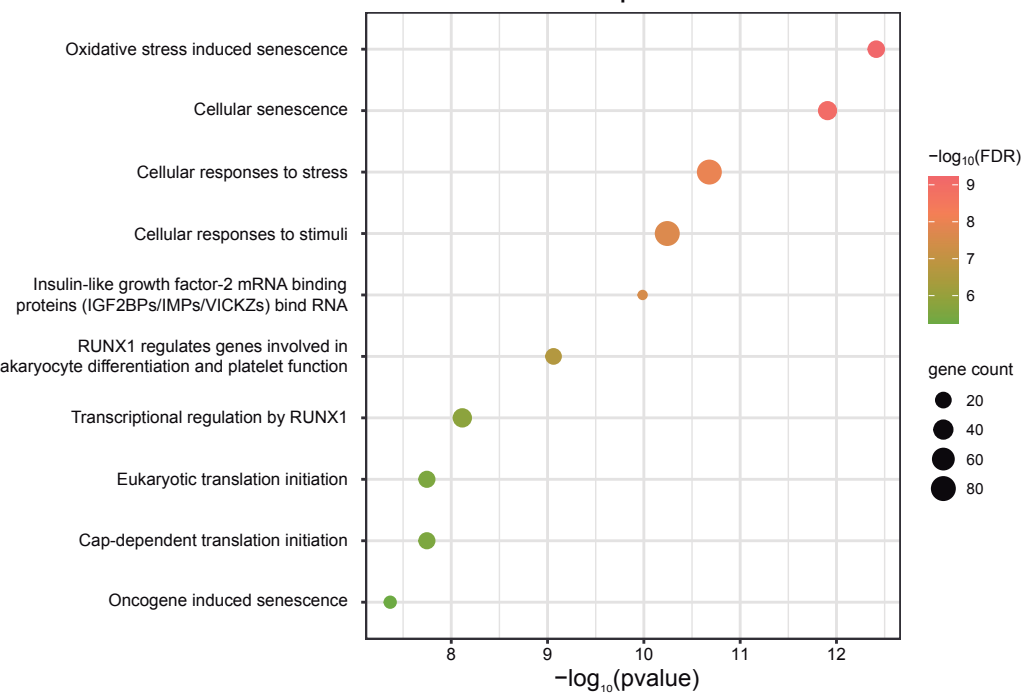

I

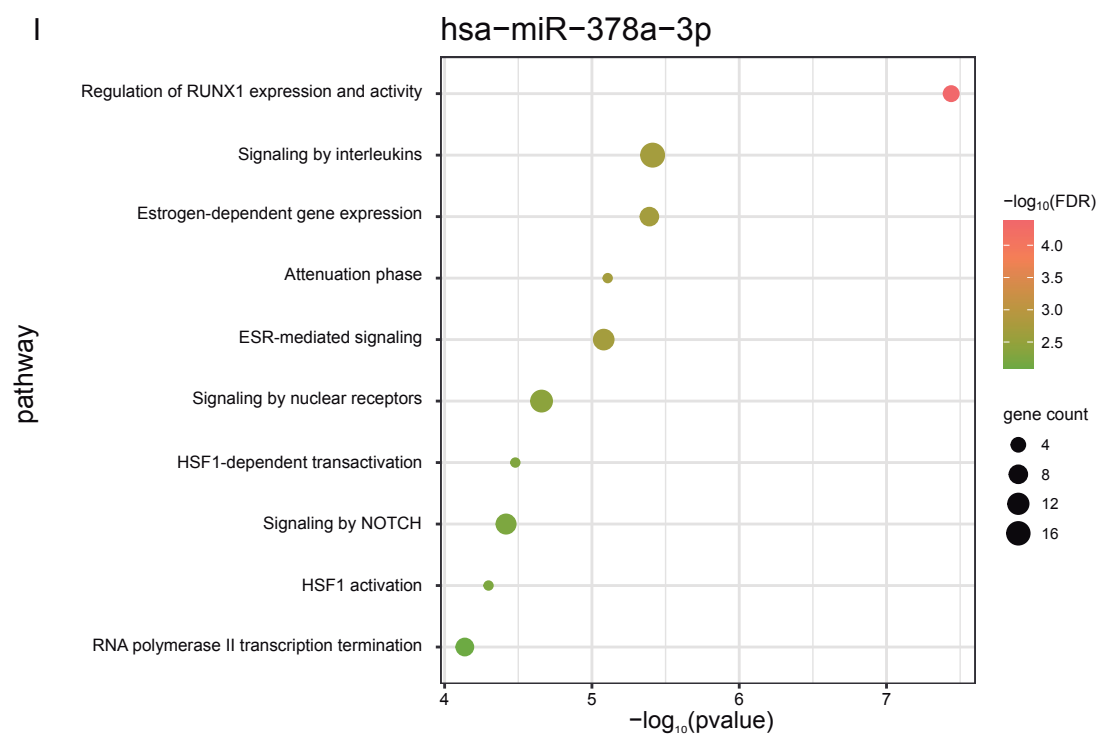

J

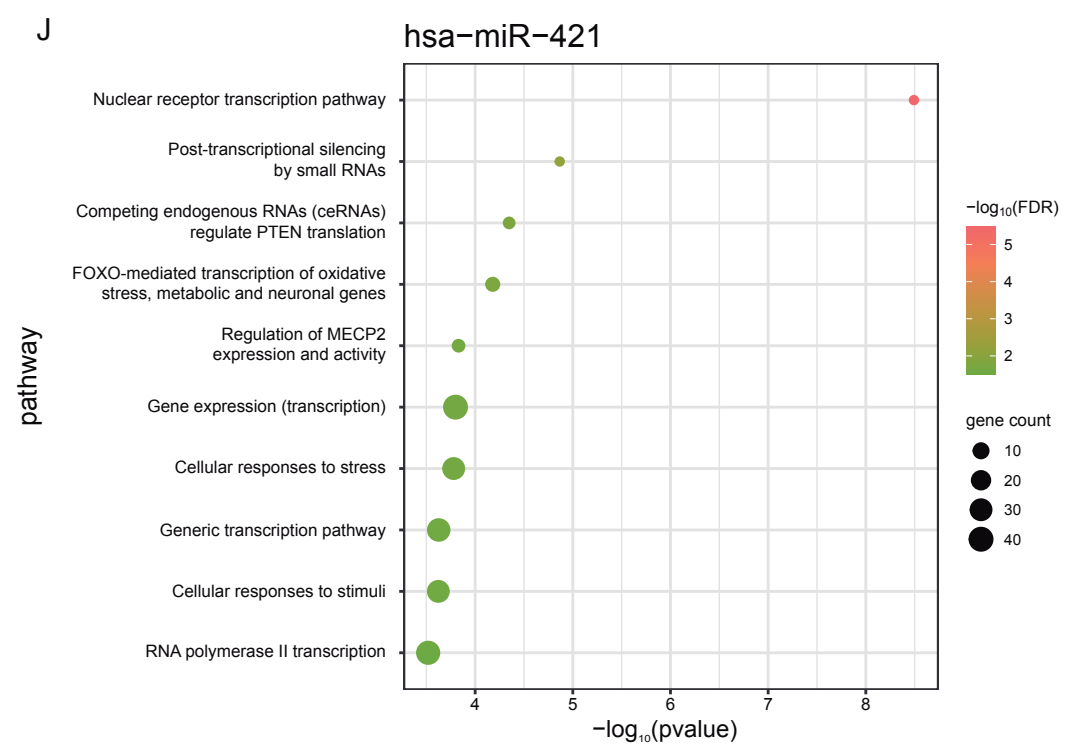

Supplement: Supplementary file 1 [file ijms-25-05467-s001.zip › Supplementary figure 1 (3).pdf]
